# Bacteriophage Cocktail for the Prevention of Multiple-Antibiotic-Resistant and Mono-Phage-Resistant *Vibrio coralliilyticus* Infection in Pacific Oyster (*Crassostrea gigas*) Larvae

**DOI:** 10.3390/pathogens9100831

**Published:** 2020-10-11

**Authors:** Hyoun Joong Kim, Jin Woo Jun, Sib Sankar Giri, Sang Guen Kim, Sang Wha Kim, Jun Kwon, Sung Bin Lee, Cheng Chi, Se Chang Park

**Affiliations:** 1 Laboratory of Aquatic Biomedicine, College of Veterinary Medicine and Research Institute for Veterinary Science, Seoul National University, Seoul 08826, Korea; hjoong@snu.ac.kr (H.J.K.); ssgiri@snu.ac.kr (S.S.G.); imagine0518@snu.ac.kr (S.G.K.); blackcat9201@snu.ac.kr (S.W.K.); kjun1002@snu.ac.kr (J.K.); lsbin1129@naver.com (S.B.L.); 2 Department of Aquaculture, Korea National College of Agriculture and Fisheries, Jeonju 54874, Korea; advancewoo@snu.ac.kr; 3 Laboratory of Aquatic Nutrition and Ecology, College of Animal Science and Technology, Nanjing Agricultural University, Nanjing 210095, China

**Keywords:** pacific oyster larvae, mass mortality, *Vibrio coralliilyticus*, bacteriophage, phage- resistant variant, cocktail phage

## Abstract

*Vibrio coralliilyticus* (*V. coralliilyticus*) is a pathogen that causes mass mortality in marine bivalve hatcheries worldwide. In this study, we used a bacteriophage (phage) cocktail to prevent multiple-antibiotic-resistant (MAR) and phage-resistant (PR) *V. coralliilyticus* infection in Pacific oyster (*Crassostrea* *gigas*) larvae. To prevent the occurrence of phage-resistant strains and decrease the effect of mono-phage treatment, we prepared a phage cocktail containing three types of *V. coralliilyticus*-specific phages and tested its prophylactic efficacy against MAR and PR *V. coralliilyticus* infection. The results of the cell lysis test showed that the phage cocktail showed an excellent bactericidal effect against the MAR and PR variants in contrast to the experimental group treated with two mono phages (pVco-5 and pVco-7). An in vivo test using Pacific oyster larvae also confirmed the preventive effect against MAR and PR variants. In conclusion, the application of the phage cocktail effectively prevented *V. coralliilyticus* infection in marine bivalve seedling production. Furthermore, it is expected to reduce damage to the aquaculture industry caused by the occurrence of MAR and PR *V. coralliilyticus*. Therefore, phage cocktails may be used for the control of various bacterial diseases.

## 1. Introduction

The marine bivalve culture industry has been the largest sector of the aquaculture industry worldwide since the development of the aquaculture industry. In 2018, it accounted for 62.7% (15,821,176 tons) of the total production of marine animals (25,233,162 tons), including fish and crustaceans [1]. Therefore, the industry for producing artificial seedlings of various marine bivalves is also actively developing. However, diseases in marine bivalve hatcheries that occur due to *Vibrio* sp. [2,3,4,5,6] and Ostreid Herpesvirus-1 [7,8,9,10,11,12] infections cause economic losses worldwide.

In marine bivalve hatcheries, *Vibrio coralliilyticus* (*V. coralliilyticus*) is one of the major causative agents of drastic mass mortality in a short period when larvae are at the free-swimming stage [4,5,13]. To control *V. coralliilyticus* infection in marine bivalve hatcheries, many studies have been conducted on the application of various antibiotics, noting problems such as the occurrence of antibiotic-resistant variants and environmental pollution due to the indiscriminate use of antibiotics in seedling production facilities [14,15].

Due to problems associated with the use of antibiotics, such as the occurrence of resistant bacteria, environmental contamination, and the killing of nonpathogenic bacteria, research has been actively performed on the development of alternative antibiotics. Among these alternative approaches is the potential prophylactic and therapeutic use of bacteriophages (phages) for the treatment of bacterial infections. In the aquaculture field, the problem of antibiotic abuse has been continuously emphasized, and alternative studies on the application of bacteriophages have been actively performed in invertebrate [16,17,18,19,20,21] and fish [22,23,24,25]. However, if the phage used also has low infectivity, it can induce phage resistance variants [26,27,28]. Therefore, the application of a phage cocktail is suggested to prevent the induction of mono-phage-resistant variants [27,29,30,31]. In addition, the use of phage cocktails is suggested to prevent various species of bacterial infections using each specific phage against the host bacteria [27,32].

The present study was aimed at preventing mass mortality in marine bivalve hatcheries, caused by multiple-antibiotic-resistant and phage-resistant *V. coralliilyticus,* using a phage cocktail. We induced a mono-phage-resistant variant. We also prepared a phage cocktail using three types of *V. coralliilyticus*-specific phages, and we evaluated the phage cocktail through in vitro and in vivo tests to confirm its prophylactic efficacy against a mono-phage-resistant variant.

## 2. Results

### 2.1. Induction of Phage-Resistant Variant

**Figure 1:**
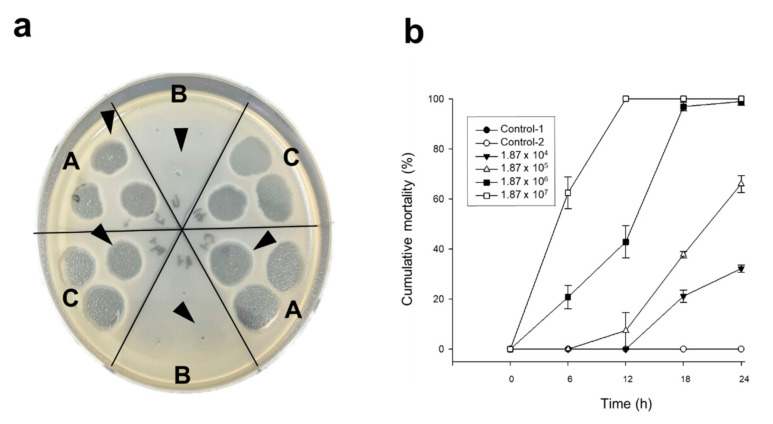
Phage-resistant variants and their pathogenicity: (**a**) pVco-7-resistant Vco58 (VcoR-7) lawn (the arrowhead indicates the zone each phage was dripped on: A indicates pVco-5, B indicates pVco-7, and C indicates pVco-14) and (**b**) pathogenicity of VcoR-7. Control-1 indicates the larvae group with filtered and sterilized seawater (FSS), and Control-2 indicates the pVco-C-treated larvae group, without VcoR-7 inoculation. Both control groups were superimposed.

Whereas pVco-7-resistant Vco58 colonies were observed, pVco-5- and pVco-14-resistant colonies were not induced under the conditions conducted in the present study (Figure 1a). Thus, we designated the pVco-7-resistant variant as VcoR-7. The induced variant was reidentified via a phage susceptibility test using other phages. The results of the pathogenicity test of VcoR-7 are shown in Figure 1b. The VcoR-7-treated group (1.87 × 10^4^ colony-forming unit (CFU)/mL) showed a cumulative mortality of 32.15% ± 1.45% after 24 h of exposure. When concentrations of 1.87 × 10^5^ CFU/mL and 1.87 × 10^6^ CFU/mL were used, each group showed a cumulative mortality of 66.03% ± 3.38% and 98.93% ± 1.5%, respectively, after 24 h. In the case of the 1.87 × 10^7^ CFU/mL-inoculated group, all larvae died within 12 h. On the other hand, only the larvae group without bacterial inoculation, which was treated only with pVco-C had a 100% survival rate. In this study, VcoR-7 also showed high pathogenicity with its parent Vco58 [16].

### 2.2. Antimicrobial Susceptibility of the Phage-Resistant Variant

The results of the antibiotic disk diffusion test are mentioned in Table 1. VcoR-7 is susceptible to Cefuroxime sodium, Imipenem, Gentamicin, Tetracycline, Ciprofloxacin, Levofloxacin, Ofloxacin Sulfonamides, Trimethoprim-sulfamethoxazole and Chloramphenicol, whereas VcoR-7 has a strong resistance to Ampicillin, Ampicillin-sulbactam, Cefepime, Cefotaxime, Cefoxitin, Ceftazidime, and Meropenem and had a weak resistance to Amoxicillin-clavulanate and Piperacillin-tazobactam and Amikacin.

### 2.3. The Bactericidal Effects of Each Phage and pVco-C against VcoR-7

**Figure 2:**
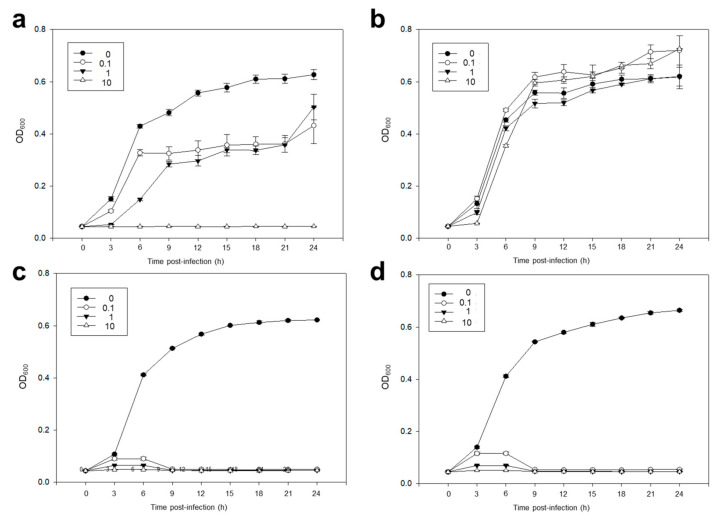
The bactericidal effects of each phage and pVco-C against VcoR-7: (**a**) pVco-5, (**b**) pVco-7, (**c**) pVco-14, and (**d**) pVco-C against VcoR-7. The *p*-value for each hour was confirmed to be <0.001, except when pVco-7 was compared to the control group; multiplicity of infection (MOI): 0.

The bactericidal effects of each phage and pVco-C against VcoR-7 are shown in Figure 2. The Optical density 600 nm (OD_600)_ value of all control groups (multiplicity of infection (MOI): 0) continuously increased during the incubation. In the case of pVco-5-treated groups, no increase in the OD value was observed in the MOI of 10, but the MOI of 0.1 and 1 showed more than 60% of the OD value of the control (MOI: 0) after 24 h (Figure 2a). In the case of pVco-7, the OD value continuously increased in the group treated with VcoR-7, similar to the control, regardless of the MOI value (Figure 2b). In contrast, pVco-14 showed a strong cell lysis effect regardless of the MOI value (Figure 2c). These results showed pVco-14 had a bactericidal efficacy against VcoR-7 similar to that previously reported for its parent strain, Vco58 [16]. pVco-C also showed a strong bactericidal efficacy in both groups infected with VcoR-7 (Figure 2d). Based on the above results, VcoR-7 showed a strong sensitivity to pVco-14 and a high titer of pVco-5. According to the results of the cell lysis test, pVco-C showed a bactericidal effect similar to that of pVco-14. VcoR-7 has a strong resistance to pVco-7. Additionally, pVco-5 showed a weak bactericidal effect when the MOI values were 0.1 and 1. These findings suggest that pVco-14 is the main contributor to the strong bactericidal effect of pVco-C.

### 2.4. Pacific Oyster Larvae Infection Prevention Using Various Concentrations of pVco-5, pVco-7, pVco-14, and pVco-C

**Figure 3:**
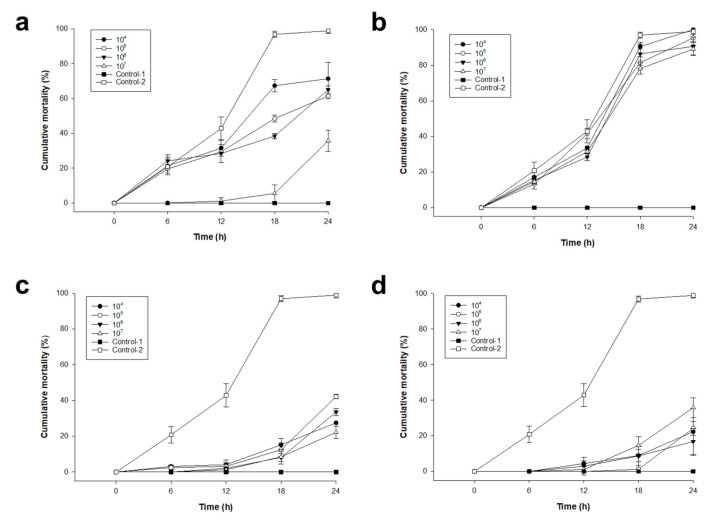
Pacific oyster larvae infection prevention using various concentrations of: (**a**) pVco-5, (**b**) pVco-7, (**c**) pVco-14, and (**d**) pVco-C. All groups except for the pVco-7-treated group had *p*-values <0.001 when compared to the control group. Control-1 indicates the phage-treated group without VcoR-7 inoculation, and Control-2 indicates the VcoR-7 inoculated larvae group without phage treatment.

In the in vivo test results of pVco-5, there was no significant difference in cumulative mortality compared to the control-2, except for the 10^7^ plaque-forming units per milliliter (PFU/mL)-treated group (Figure 3a). In addition, pVco-7 showed no protective effect on the infection with VcoR-7 (Figure 3b). Meanwhile, pVco-14 and pVco-C showed less than 40% cumulative mortality in various concentrations in phage-treated groups (Figure 3c,d). The results of the in vivo test were remarkably similar to those of the in vitro test. The utilization of a phage cocktail, which aimed to compensate for the disadvantage of using a single phage, showed strong preventive effects in in vitro and in vivo tests.

## 3. Discussion

The emergence of multiple-antibiotic-resistant bacteria continues to be a problem due to the misuse of various antibiotics in aquaculture industries. As a result, limiting the application of antibiotics in this industry is becoming an urgent need. Thus, the aim of several studies in this field is the identification and development of alternative strategies, such as vaccination [33], and the use of bacteriophages [34], probiotics [35], and immunostimulants [36]. Vaccination is an ideal method to prevent diseases caused by pathogen infections, but it cannot be applied to invertebrates because of the absence of an acquired immune system. Therefore, the phage application is actively studied to prevent bacterial infection in invertebrates and has shown a substantial prophylactic efficacy [16,17].

However, phage-resistant strains may also be induced. In fact, the pVco-7-resistant variant induced in our study did not show sensitivity to pVco-7 in the in vitro and in vivo tests. In the present study, pVco-C showed bactericidal effects similar to those of the pVco-14 mono-phage treatment. This suggests that pVco-14 is the most effective at lysing VcoR-7. The pVco-14-resistant variant was not induced in this study. However, it could be speculated that the advantage of using pVco-C, instead of pVco-14, is superior due to the fact that it could prevent a possible emergence of single-phage-resistant mutants after the long-term usage of a mono-phage. Therefore, a phage cocktail could be utilized to prevent the emergence of phage-resistant bacteria that may occur when a mono-phage is applied. pVco-5, pVco-7, and pVco-14 have been reported to inhibit Vco58 and other *V. coralliilyticus* strains at 27 °C [16,17], indicating that the three phages are suitable for bivalve hatchery environments.

In general, a phage cocktail can be utilized not only for the prevention and treatment of various bacterial infections but, also, for the control of phage-resistant bacteria that may occur during mono-phage application [26]. In the present study, we used a phage cocktail to prevent the occurrence of phage-resistant variants and confirmed the bactericidal effects of pVco-C in in vitro experiments. The phage resistance mechanisms of bacteria have been widely studied, including the prevention of phage adsorption, blocking of phage receptors, production of extracellular matrix, and production of competitive inhibitors [30]. The use of cocktail phages can arise a competition for the same receptor in each phage. If there is the same infection mechanisms between used phages, phage cocktails do not show infectivity against the mono-phage-resistant strain. In the present study, pVco-5 and pVco-14 showed a bactericidal effect against the pVco-7-resistant variant. It means that both phages do not show the same mechanisms to infect the host cell in comparison with pVco-7. Therefore, it is suggested that the infectious mechanism of each phage used in preparation of phage cocktails should be considered.

## 4. Materials and Methods

### 4.1. Bacterial Strain and Growth Conditions

*V. coralliilyticus* 58 (designated Vco58), a highly virulent strain against Pacific oyster (*Crassostrea gigas*) larvae [5,16], was used as a host for phage isolation and propagation. Sodium chloride (final concentration 2.0%) supplemented with tryptic soy agar (TSA; BD Difco, Sparks, MD, USA), tryptic soy broth (TSB; BD Difco, Sparks, MD, USA), and semi-solid TSB top agar were used for bacterial growth and phage propagation. Vco58 and phage-resistant strains were cultured in an incubator at 27 °C for 24 h.

### 4.2. Phage Cocktail Preparation

**Figure 4:**
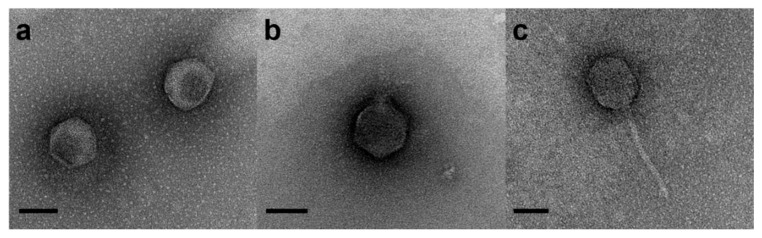
Phages used in this study: (**a**) pVco-5, (**b**) pVco-7, and (**c**) pVco-14. Bar indicates 50 nm.

Three previously isolated *V. coralliilyticus* specific phages, pVco-5 and pVco-7 [17] and pVco-14 [16], were used to prepare the phage cocktail (designated pVco-C) (Figure 4). All phages were confirmed as lytic phages through the whole-genome sequence analysis [37], unpublished data. Phages were purified using the Caesium chloride density gradient method [38] and adjusted to a concentration of 10^9^ plaque-forming units per milliliter (PFU/mL) and mixed in a ratio of 1:1:1.

### 4.3. Induction of Phage-Resistant Variants of V. coralliilyticus

Ten milliliters of each pure phage suspension (≥10^8^ PFU/mL) were mixed with 100 mL of Vco58 cultured in TSB (10^6^ CFU/mL). After gentle blending, the mixture was incubated in a shaking incubator at 27 °C and 150 rpm for 24 h. One hundred microliters of cultured lysates were mixed with 3 mL of TSB top agar. After gentle mixing, the cells were spread onto TSA and incubated at 27 °C for two days. If colonies occurred on the plate, they were re-cultured in TSB at 27 °C and 150 rpm for 12 h. Then, 100 µL of the bacterial suspension was spread onto a TSA plate. Ten microliters of the previously inoculated phage (≥10^9^ PFU/mL) were subsequently dripped onto the bacterial lawns and incubated at 27 °C for 24 h. If phage plaques were not observed, the spot assay using the strains that did not form plaques were repeated, and cultures that produced no plaque were chosen as phage-resistant strains.

### 4.4. Antimicrobial Susceptibility of the Phage-Resistant Variant

The antimicrobial susceptibility test of the pVco-7-resistant strain was carried out using 21 antibiotics by using the disk-diffusion method following the Clinical and Laboratory Standard Institute (CLSI) recommendations [39]. The used antibiotic disks are listed in Table 1. A standard disk diffusion test was performed on Muller Hinton Agar (BD Difco, Sparks, MD, USA). With the exception of temperature, all experimental conditions were performed following the CLSI guidelines. The antimicrobial susceptibility test was not conducted at 35 ± 2 °C, as suggested by CLSI, but at 27 °C, the optimal growth temperature of *V. coralliilyticus* and the incubation temperature of Pacific oyster larvae. The results were interpreted according to CLSI guidelines. *Escherichia coli*, ATCC 25922, was used as the quality control strain.

### 4.5. Pathogenicity of the Phage-Resistant Variant

Five-day-old healthy Pacific oyster larvae (100–140 µm) were used to confirm the pathogenicity of the phage-resistant variant. A challenge test was performed as previously described by Kim et al. [4]. Oyster larvae (*n* = 50 ± 13) were placed into each well of a 6-well cell culture plate (SPL, Pocheon, Korea) with 10 mL of 32 practical salinity units (psu) of filtered and sterilized seawater (FSS). The phage-resistant variant was inoculated into each well, and the final concentrations were adjusted to 0, 1.87 × 10^4^, 1.87 × 10^5^, 1.87 × 10^6^ CFU, and 1.87 × 10^7^ CFU/mL, and incubated at 27 °C for 24 h. To evaluate the toxicity of the phage cocktail, the pVco-C-treated group without bacterial inoculation was also used. The cumulative mortality of each well was monitored at 6-h intervals using an inverted microscope BX41 (Olympus, Tokyo, Japan). The pathogenicity tests were performed in triplicate.

### 4.6. Bacterial Cell Lysis Test of the Phage-Resistant Variant

To evaluate the bactericidal effect of each phage and pVco-C, the phage-resistant variant induced in this study was used as the host. Bacterial cell lysis tests were performed as previously described by Kim et al. [17]. The multiplicity of infection (MOIs) of each phage and pVco-C was regulated to 0, 0.1, 1, and 10. The absorbance (OD_600_) of each group was checked for 24 h at 3-h intervals. All tests were conducted in six replicates.

### 4.7. Prophylactic Efficacy of the Phages and Phage Cocktail

For the in vivo tests, phage preparations were partially purified using 10% polyethylene glycol 8000, 1-M sodium chloride, and FSS, as previously described by Kim et al. [16]. Five-day-old healthy Pacific oyster larvae (*n* = 50 ± 19) were placed into each well of a 6-well cell culture plate with 10 mL of each purified phage suspension, adjusted to 10^4^, 10^5^, 10^6^, and 10^7^ PFU/mL and acclimated at 27 °C for 1 h. After adaptation, 10^6^ CFU/mL of the phage-resistant variant solution, washed with FSS three times, was inoculated into each well and incubated for 24 h at 27 °C. The cumulative mortality was checked for 24 h at 6-h intervals. We also used two control groups, one without a phage and one without bacteria. Larvae without cilia and intravalvular movement were considered dead following the protocol previously described by Sugumar et al. [5]. The in vivo test was performed in triplicate under the same conditions.

### 4.8. Statistical Analysis

Statistical analysis was conducted using the SigmaPlot 14.0 software (Systat Software, Inc. Chicago, IL, USA). One-way analysis of variance (ANOVA) was used to analyze the data followed by the Bonferroni post-hoc test. A *p*-value < 0.05 was considered statistically significant.

## 5. Conclusions

Like antibiotic-resistant variants, phage-resistant strains can also be induced in nature. In the present study, the pVco-7-resistant variant was induced and shown to have high pathogenicity towards Pacific oyster larvae as its parent strain. We prepared a phage cocktail to confirm its prophylactic efficacy against a mono-phage-resistant variant. The results show that this phage cocktail was found to be effective in the prevention of the mono-phage-resistant *V. coralliilyticus* infection in Pacific oyster larvae. This study may contribute to the reduction of the damaging effects, caused by the occurrence of multi-drug-resistant variants, to the aquaculture industry. Moreover, the use of a phage cocktail may prevent the emergence of phage-resistant bacteria that can occur during single-phage therapy and could potentially be used to control various bacterial diseases.

## Figures and Tables

**Table 1:** Result of the antimicrobial susceptibility test of the pVco-7-resistant *Vibrio coralliilyticus* strain (VcoR-7).

Antimicrobial Agent	Zone Diameter (mm)Interpretive Criteria			VcoR-7	Antimicrobial Agent	Zone Diameter (mm)Interpretive Criteria			VcoR-7
	S *	I *	R *	Zone Diameter/Criteria		S	I	R	Zone Diameter/Criteria
Ampicillin	≥17	14–16	≤13	0/R	Imipenem	≥23	20–22	≤19	26/S
Amoxicillin-clavulanate	≥18	14–17	≤13	16/I	Meropenem	≥23	20–22	≤19	18/R
Ampicillin-sulbactam	≥15	12–14	≤11	11/R	Amikacin	≥17	15–16	≤14	15/I
Piperacillin	≥21	18–20	≤17	0/R	Gentamicin	≥15	13–14	≤12	15/S
Piperacillin-tazobactam	≥21	18–20	≤17	20/I	Tetracycline	≥15	12–14	≤11	20/S
Cefepime	≥25	19–24	≤18	18/R	Ciprofloxacin	≥21	16–20	≤15	25/S
Cefotaxime	≥26	23–25	≤22	21/R	Levofloxacin	≥17	14–16	≤13	25/S
Cefoxitin	≥18	15–17	≤14	14/R	Ofloxacin	≥16	13–15	≤12	22/S
Ceftazidime	≥21	18–20	≤17	15/R	Trimethoprim-sulfamethoxazole	≥16	11–15	≤10	27/S
Cefuroxime sodium	≥18	15–17	≤14	19/S					

* S: susceptible, I: intermediate, and R: resistant.
